# Risk factors of professional burnout for nurses, health technicians and midwives at the beni mellal regional hospital, Morocco

**DOI:** 10.1192/j.eurpsy.2023.665

**Published:** 2023-07-19

**Authors:** R. Ilham, H. khalki, S. Boulbaroud, F. Azzaoui

**Affiliations:** ^1^Biology and Health, Faculty of Science, Ibn Tofail University, Kenitra, Morocco; ^2^Polydisciplinary Faculty, Sultan Moulay Slimane University, Beni Mellal, Morocco

## Abstract

**Introduction:**

Burnout is a topical issue, which concerns all fields and more particularly our health field.

**Objectives:**

Our descriptive study aims to evaluate the prevalence of burnout and describe its risk factors among nurses, health technicians and midwives in the regional hospital of Beni Mellal. It is being carried out from February to June 2022, with 113 participants.

**Methods:**

Given the nature of our research, the data collection tool consists of two questionnaires, the first to study personal, professional data and risk factors for burnout, and the second to assess burnout among our participants, based on the MBI in its French version.

**Results:**

Our study showed that burnout affected more than three quarters of our sample, 59.3% of them had high emotional exhaustion, 26.5% had high depersonalization and 41.6% had low personal accomplishment.

The occurrence of this syndrome was the result of several risk factors, the most frequent being: stress related to the Covid-19 pandemic, poor organization and management of services, insufficient means and personnel, lack of recognition and motivation, unsatisfactory salary/effort, degraded interpersonal relations and confrontation with suffering.

**Conclusions:**

In conclusion, burnout is a palpable reality among nurses, health technicians and midwives.Our alarming results must lead to the implementation of preventive actions while insisting on the organization of work and the valorization of the Moroccan caregiver.

**Disclosure of Interest:**

None Declared

